# Frozen moments: flashback memories of critical incidents in emergency personnel

**DOI:** 10.1002/brb3.325

**Published:** 2015-05-13

**Authors:** Birgit Kleim, Martina-Barbara Bingisser, Maren Westphal, Roland Bingisser

**Affiliations:** 1 Department of Experimental Psychotherapy, University of ZurichZurich, Switzerland; 2 Department of Psychiatry, Psychotherapy and Psychosomatics, University Hospital ZurichZurich, Switzerland; 3 Department of Emergency Medicine, University Hospital BaselBasel, Switzerland; 4 Department of Psychology, Pace UniversityPleasantville, New York; 5 New York State Psychiatric Institute, Columbia UniversityNew York, New York

**Keywords:** Anxiety, burnout, depression, emergency department, emotional memory, flashback, intrusion

## Abstract

**Background:**

Emergency Department personnel regularly face highly stressful situations or critical incidents (CIs) that may subsequently be recalled as unbidden intrusive memories. In their most extreme form, such memories are reexperienced as if they were happening again in the present, as flashbacks. This study examined (1) which CIs are associated with flashback memories; (2) candidate person and work-related features that predict flashback memories; and (3) the association between flashback memories and anxiety, depression, and emotional exhaustion.

**Methods:**

Emergency nurses (*N* = 91; 80.2% female) were recruited from two urban teaching hospitals and filled in self-report questionnaires.

**Results:**

A majority (*n *=* *59, 65%) experienced intrusive memories; almost half of the sample reported that their memories had flashback character. Those involved in resuscitations in the past week were at a fourfold risk for experiencing flashbacks. Having worked more consecutive days without taking time off was associated with a somewhat lower incidence of flashbacks. Moreover, older individuals who reported more work-related conflicts were at greater risk for experiencing flashback memories than their younger colleagues with heightened work conflict and flashback memory scores, respectively. Flashback memories were associated with heightened symptoms of anxiety, depression, and emotional exhaustion.

**Conclusions:**

The present findings have implications for evidence-based health promotion in emergency personnel and other individuals regularly exposed to CIs.

## Introduction

Emergency Department personnel regularly face a variety of challenging situations or “critical incidents” (CI) such as being confronted with the death of patients, including young children, assisting victims of violent crimes and traffic collisions, and witnessing failed resuscitation attempts. Such CIs may subsequently be recalled as unbidden, intrusive memories, that tend to occur mostly in perceptual form, such as images or “frozen moments”, but they can also consist of verbal thoughts, sounds, smells and tastes (Ehlers et al. 2004). In their most extreme form, they are reexperienced as “flashbacks”, that is, a very intense reliving of traumatic events as if they were happening again in the present moment. Participants in the present study reported reexperiencing, for instance, a situation where distressed parents were present when resuscitation attempts of their 4-year-old daughter were terminated, or treating a patient with 80% burn injuries, or a patient speaking about their upcoming holidays, who died 5 min later. While such intrusive memories may occur in healthy individuals in the wake of stressful incidents, flashbacks and persistent distressing intrusions are a hallmark symptom of posttraumatic stress disorder (PTSD). Intrusions may appear “out of the blue”, in very different contexts than the original incident, tend to be highly distressing, and may persist for years as prominent symptoms of many types of psychopathology, such as depression or anxiety (Brewin et al. 2010).

From a public health perspective, a better understanding of intrusions is important, as experiencing intrusions could impact work performance, lead to absenteeism, and may eventually evolve into long-term disability in populations repeatedly exposed to CIs such as emergency personnel (Laposa et al. 2003). Considering the growing role in hospital admissions and the heavy workload of EDs (Schuur and Venkatesh 2012), the question arises as to how we can support those who experience CI flashback memories and how such unbidden memories may be prevented. Very few studies have investigated intrusive memories and flashbacks of CIs in emergency personnel. Half of the ambulance workers in one study endorsed symptoms of intrusive memories and nearly all of the intrusions were about incidents involving the death of another person, often including children (Alexander and Klein 2001). Other studies identified distressing CIs, such as caring for a patient who is a relative or close friend and is dying or in a serious condition, caring for a child victim, or threatened physical assault of self as most upsetting events (Clohessy and Ehlers 1999; Declerque et al. 2011).

The above studies did not address the question of which CIs are subsequently reexperienced as intrusive memories. It is possible that some CIs are more psychologically “toxic” than others, in which case early recognition and targeted interventions might be useful to prevent adverse long-term psychological effects of such events. Another unaddressed question is the relative contribution of work-related stressors and person-related factors to the prevalence of intrusive memories among emergency personnel. Work volume, patient load, number of nightshifts, and number of consecutive work days since last taking time off are work features that have been associated with stress reactions and burnout in medical professions (Alimoglu and Donmez 2005; Rosenstein 2013) and thus may present important risk factors for the emergence and maintenance of intrusive memories. In emergency personnel, time pressure and shiftwork, working full-time as compared to part-time and not taking time off work may contribute to overall stress levels and thus lead to greater likelihood of experiencing intrusive memories (Clohessy and Ehlers 1999). In terms of person-related factors, previous studies have linked both younger (Alimoglu and Donmez 2005), as well as older age (Streu et al. 2013) to increased work stress and burnout in medical professions, or have found no association between age and burnout (Goldberg et al. 2012). A consistent finding, however, has been that females endorse higher rates of stress and stress-related psychopathology, such as posttraumatic stress disorder or depression (Nolen Hoeksema et al. 1999; Tolin and Foa 2006). Alcohol has also been implicated as a likely contributor to stress and burnout in medical professions (Hochberg et al. 2013). Another open question is whether and how person- and work-related factors interact in increasing risk of developing intrusive CI memories. For instance, female or older emergency personnel reporting more frequent resuscitation situations or greater workloads may be especially prone to developing intrusions. Due to the mixed findings on the association of age on psychological symptoms in the context of work- stress, we explored interaction hypotheses with regards to age, but had no directional hypotheses. Finally, it is important to know about the psychological toll such memories might exact. Specifically, anxiety, depression, and emotional exhaustion are features of burnout that have been frequently reported by emergency personnel (Declerque et al. 2011). Intrusive memories may be key factor involved in the evolution and maintenance of such symptoms.

Empirical answers to these three interrelated questions could help screen emergency personnel vulnerable to experiencing flashback memories of CIs and could assist in developing prevention and intervention programs. First, the present study examined prevalence, characteristics and content of intrusive memories and their flashback characteristic in a sample of emergency department nursing personnel. Second, we investigated the influence of person- (e.g., age, sex, relationship status, alcohol consumption) and work-related features (e.g., working predominantly day vs. night shifts and part vs. full-time, time since last taken days off work, critical resuscitation experiences in the past week, conflicts at work, such as arguments, disagreements or direct confrontations with colleagues or patients) on intrusive memories. Relatedly, we investigated the association between selected interactions between age, sex and work-related variables predict the experience of CI flashbacks. Third and finally, we examined associations between flashback memory experiences and symptoms of anxiety, depression, and emotional exhaustion and predicted greater symptoms of psychopathology in those who experienced CI flashbacks compared to those who did not.

## Methods

### Participants and procedures

The local ethical review boards approved the study. We examined prevalence, content and characteristics of intrusive memories related to CIs in a two-site study on 91 nurses (80.2% female) employed in the Accident and Emergency Department of two urban teaching hospitals. Both departments are staffed with 94 and 91 nurses, respectively. Forty-nine percent agreed to participate in the study and answered questions about content and characteristics of intrusive memories of CIs and completed a questionnaire comprising demographic and work-related questions, as well as questions regarding anxiety and depressive symptoms and burnout. The sample was representative of emergency nurses in the two hospitals in terms of sex, age, and time since employment, see Table 2001 for sample characteristics. Data were collected via anonymous questionnaires.

**Table 1:** Demographic and work-related sample characteristics (*N *=* *91)

	*n*	%
Sex		
Male	18	19.8
Female	73	80.2
Education		
Secondary school degree	43	47.3
High school degree	17	18.7
College/University	29	31.9
Undisclosed information	2	2.2
Relationship status		
In relationship	23	25.3
No current relationship	68	74.7
Work volume		
Full-time	31	34.1
Part-time	60	65.9
Current medication		
Yes	23	25.3
No	68	74.7
Age, M (SD)	41	(9.66)
Alcohol units past week, M (SD)	2.62	(3.45)
Nightshifts per week, M (SD)	2.48	(1.77)
Resuscitations last week, M (SD)	1.30	(2.10)
Days since last taken time off work, M (SD)	9.88	(10.28)
Conflict at work during the past week, M (SD)	0.86	(0.82)

### Measures

Demographic and work-related factors were assessed with single items, indexing age, sex, alcohol consumption, volume of work, whether participants worked predominantly day versus night shifts, patient loads during a regular shift, time since last taken days off work, number of resuscitation and shock room experiences during the past week. We also assessed with four items whether participants had experienced any work conflicts with colleagues, medical doctors, other emergency staff, or patients, and with one item whether participants had been involved in difficult work-related decisions in the past week.

**Figure 1:**
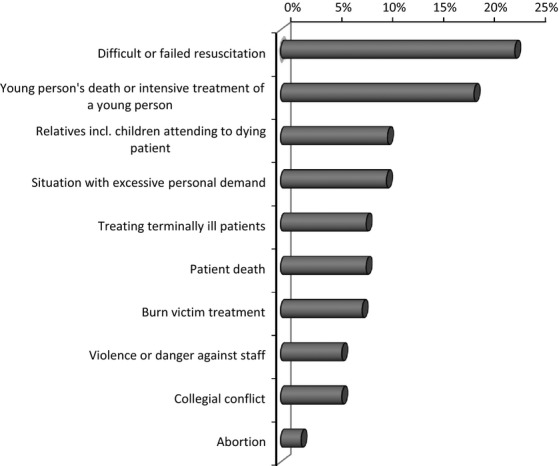
Critical incidents experienced as intrusive memories in the past week (in %). Note: Based on *n *=* *51 memories reported by respondents; five nonmemories were excluded.

Intrusive memories were assessed by a questionnaire adapted from Hackmann et al. (2004) This questionnaire assesses whether participants experience intrusive work-related memories (yes or no). Further items index the frequency with which intrusive memories occur during the past week on a scale from 0 (not at all) to 3 (daily), as well as sensory components (whether the intrusion occurs in form of thoughts, feelings or other sensory aspects) and distress related to the intrusive memory indexed as either not at all, considerable, or extreme. Participants were also asked to describe their most frequent intrusive memory. One item indexed flashback quality of the memory, that is, whether the memory appears to be happening again in the present moment rated on a scale from 0 (not at all) to 3 (absolutely). This score was used to construct a score indicating whether participants experienced flashbacks (score 1–3) or not (score 0). Memory content was later scored by independent raters and categorized into 10 categories (see Fig.1: difficult or failed resuscitation, young person's death or difficult treatment of a young person, relatives incl. children attending to dying patient, treating terminally ill patients, patient death, burn victim treatment, violence or danger against staff, abortion, collegial conflict, situation with excessive personal demand) with high interrater reliability (κ = 0.79).

Anxiety and depression was assessed with the 14-item Hospital Anxiety and Depression Scale (HADS (Zigmond and Snaith 1983)). Each item is scored from 0 to 3, resulting in scores between 0 and 21 for either anxiety or depression, with good internal consistency in the present study, Cronbachs *α* = 0.68 (for depression) and *α* = 0.78 (for anxiety).

Emotional exhaustion was assessed with the Maslach Burnout Inventory, (MBI (Maslach et al. 1986)). The scale has 22 items that are answered on 6-point Likert scales and index the cumulative effects of work-related pressures on emotional exhaustion, that is, depletion of emotional resources, being overextended and emotionally drained. The scale contains nine items and showed very good internal consistency in the present study, Cronbachs *α* = 0.84.

### Data analysis

Data were analyzed using SPSS for Windows, version 20.0. (IBM, Armonk, NY) Descriptive statistics were used to describe intrusive memory and flashback frequency. We examined the association between specific demographic and work-related features and experiencing flashback memories using logistic regression. Additionally, we tested whether interactions between age, sex and significant work-related variables significantly contributed to explaining the occurrence of flashback memories. Significant interactions were interpreted by plotting the effects and differences between simple slopes using standardized variables. Finally, we examined in a multivariate general linear model whether those who experienced flashback memories had higher levels of anxiety and depression symptoms and emotional exhaustion compared to those without flashback memories.

## Results

### Prevalence, characteristics and content of intrusive CI memories

Most participants in our study (*n *=* *59, 65%) experienced intrusive memories of CIs. Forty-two participants (46% of the total sample) reported experiencing flashback memories, that is, memories perceived as happening in the present. Whereas 33 individuals (36% of total sample) reported no distress associated with intrusive memories, 40 individuals (44% of total sample) experienced considerable distress related to intrusive memories, including four individuals reporting extreme distress (4%).^1^ Thirty-six individuals (40% of the total sample) reported experiencing intrusive memories up to three times during the past week, two individuals (2%) experienced the memory on a daily or almost daily basis. Figure1 depicts intrusive memory content based on 51 reported memories (not all participants disclosed the content of their memories), which included difficult or failed resuscitations (22.9%), followed by young persons’ deaths or intensive care (18.8%) and treating terminally ill patients or severe injuries (8.4%). Further memories included patient's death, relatives attending to a dying patient, situations characterized by excessive personal demands, treatment of burn victims, violence or danger against hospital staff, and conflicts with colleagues Table 2001.

### Prediction of flashback memories by person- and work-related characteristics

Neither age, sex, relationship status nor alcohol/drug use predicted the experience of flashback memories in the total sample, *χ*² = 2.64, df = 5, *P *=* *0.756, all *P* values > 0.430. Candidate work-related factors did, however, significantly predict the experience of flashback memories, *χ*² = 14.04, df = 7, *P *=* *0.050. Significant predictors were involvement in critical resuscitation situations in the past week, OR = 3.53; 95% CI 1.05–11.94; *P *=* *0.042, number of consecutive days at work since last taking time off, OR = 0.93; 95% CI 0.86–1.0; *P *=* *0.041 and work conflicts during the past week, OR = 0.23; 95% CI 0.07–0.85; *P *=* *0.023. Working full- or part-time, working nightshifts, patient load during a normal shift, and being involved in difficult decisions in the past week were not significant predictors of experiencing flashback memories, all *P* values > 0.341.

**Figure 2:**
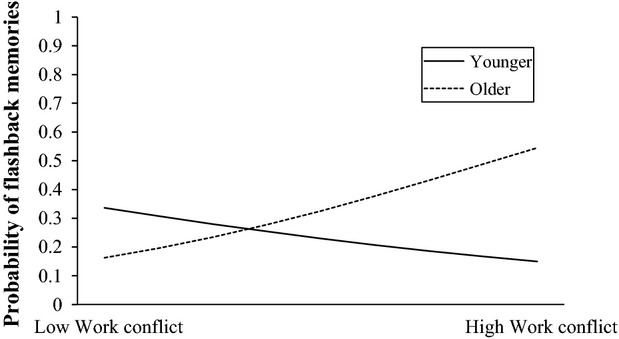
Age and work conflict interact in predicting flashback experiences: Older nurses with high levels of work conflicts showed the greatest probability of reporting flashback memories. Note: Slopes depicting groups of younger and older participants were plotted at 1 SD above and below the mean age of the sample.

Interactions were calculated for the three significant work-related predictors and sex and age, respectively, hence resulting in six interaction models. The interaction between age and work conflicts was significant over and above the respective main effect, OR = 0.1.07; 95% CI 1.04–1.14; *P *=* *0.037). The remaining interactions including age, sex and work-related variables were nonsignificant. Follow-up analyses revealed that age moderated the effects between work conflicts and flashback occurrence. More specifically, having been involved in work conflicts during the past week increased the likelihood of experiencing flashback memories by more than 10 times in older, OR = 10.89; 95% CI 1.99–59.72; *P *=* *0.006, but not in younger ED personnel, OR = 0.63; 95% CI 0.13–2.99; *P *=* *0.625. Figure2 plots simple slopes of younger and older nurses and depicts that older nurses with high levels of work conflicts showed the greatest probability of reporting flashback memories.

### Flashback memories and anxiety, depression and emotional exhaustion

As shown in Table 2005, those who experienced CI flashback memories reported significantly more anxiety and depression symptoms than those who did not endorse such symptoms, as well as more emotional exhaustion.

**Table 2:** Group differences from multivariate regression analysis predicting anxiety, depression symptoms, and emotional exhaustion based on experiencing flashback memories (*N* = 74)

Variable	Flashbacks (*n *=* *27)	No Flashbacks (*n *=* *47)	Sign. differences
HADS Anxiety, Mean (SD)	5.78 (3.29)	3.85 (2.66)	*F* (1, 73) = 7.56*
HADS Depression, Mean (SD)	4.07 (3.35)	2.57 (2.94)	*F* (1, 73) = 4.03*
MBI Emotional exhaustion, Mean (SD)	24.14 (8.80)	15.11 (7.80)	*F* (1, 73) = 20.95***

Note: HADS = Hospital Anxiety and Depression Scale, range anxiety subscale 0–21, range depression subscale 0–21, with scores greater than 7 indicating borderline symptomatology; MBI = Maslach Burnout Inventory, range emotional exhaustion scale 0–54, with scores between 19 and 27 indicating moderate burnout

*** *P *<* *0.001

* *P *<* *0.05.

## Discussion

Intrusive memories of CIs were common and distressing experiences among ED nursing personnel. More than 60% reported intrusive memories and almost half of the sample experienced memories of work-related CIs with flashback quality. The odds of having flashback memories were almost four times greater in those individuals involved in critical resuscitations in the past week compared to those who were not. Having worked more consecutive days since last taking time off was associated with lower flashback experience. Age moderated the relation between work conflict and flashback experiences. Older nurses who had experienced recent work conflicts were at higher risk for experiencing flashbacks than their younger colleagues without work conflicts. Overall, experiencing flashback memories was associated with greater levels of anxiety, depression, and emotional exhaustion.

The finding that 30% of ED nurses endorsed flashback memories is in line with previous studies of intrusive memories, although these did not always assess intrusions experienced as more intense flashback memories (Clohessy and Ehlers 1999; Laposa and Alden 2003; Embriaco et al. 2012). The most frequent intrusive memories in our sample were about difficult or failed resuscitations, followed by young persons’ deaths or their intensive or resuscitation treatment, relatives attending to a dying patient and, on a more general level, situations imposing excessive personal demand on staff, such as attending to several patients at the same time, having to make crucial decisions and questioning their appropriateness later. These situations require attention and evidence-based interventions. Following such incidents, staff members may benefit from discussing incidents amongst each other or with a supervisor or designated third person of trust (Healy and Tyrrell 2013; Tucker and Scott 2013). Peer support may play an important role in preventing long-term psychological distress by normalizing and alleviating symptoms of distress (Dowling et al. 2006; Strand et al. 2010). Encouraging staff to write about CIs may also be helpful given research demonstrating positive health effects of writing about stressful events (Niles et al. 2014), including a study showing that expressive writing ameliorated the evolution of intrusive thoughts (Lepore 1997). Such expressive writing may help to elaborate and better understand the incident and be mentally prepared for the occurrence of subsequent intrusive memories or it may help by desensitizing individuals to intrusive thoughts (Lepore 1997) and buffering against rumination (Sloan et al. 2008).

Involvement in critical resuscitation situations during the past week was associated with a fourfold increased probability of experiencing flashbacks. This suggests that exposure to resuscitation may have a significant influence on emotional memory formation and psychological well-being in ED personnel and merits further empirical investigation. For example, future studies could examine a possible dose–response relationship between repeated exposure to such situations and accompanying emotional distress. ED staff involved in such critical resuscitations may benefit from debriefing of resuscitation situations and other interventions designed to facilitate postevent processing and staff support (Healy and Tyrrell 2013; Tucker and Scott 2013). Having worked more consecutive days since last taking time off was associated with a lower odds of experiencing flashback experience. This could reflect a possible bias, however, in that those who experience flashback memories and possibly other symptoms of stress and psychopathology might be more likely to take time off. Sex and alcohol were not significant predictors of flashback experiences, but this may mainly be due to a restriction of range in the present sample, which consisted predominantly of female individuals and low alcohol consumption. Interestingly, age had an important influence on the relationships between experiencing work conflicts and flashback experience. Older personnel seemed to be at heightened flashback risk in the context of having experienced conflicts at work, which was associated with a 10-fold higher risk of flashbacks. Older staff members under such strenuous conditions appear to be particularly vulnerable to develop flashback memories and constitute a target group for screening and prevention programs, given the association between experiencing flashbacks and symptoms of burnout such as emotional exhaustion observed in the current study. There are several possible explanations for the increased vulnerability in older ED personnel in the context of conflict at work. Older personnel may be trained differently than younger cohorts, who may be more prepared and thus better equipped for specific stressful situations. Moreover, there may be a biological component, as aging may wear out the systems in the brain responsible for adaptive stress responses and thus make older individuals more vulnerable to respond to stress under such conditions (Sapolsky et al. 2002). Further prospective longitudinal data will be crucial to clarify these relationships.

As one might expect, flashback memories were associated with elevated anxiety and depression and higher levels of emotional exhaustion, a core component of burnout often reported by ED workers. These findings underline the dysfunctional nature of such memories and their possible contribution to work-associated psychopathology.

The current study has a number of limitations. First, the cross-sectional nature of our investigation precludes conclusions about causal associations. Associations between intrusive memories and psychopathology could be explained by intrusions triggering symptoms of depression, anxiety, or emotional exhaustion. Alternatively, depression, anxiety, or emotional exhaustion might lead to the experience of intrusive memories. Second, our sample is limited to nurses and the results need to be replicated in other groups, such as medical staff or ambulance workers. This might be particularly relevant in the case of age as moderating variable as suggested by conflicting findings regarding the role of age in the literature on burnout in health professionals, with some studies finding that younger age was associated with greater risk among nurses (Alimoglu and Donmez 2005) while higher age was associated with higher burnout risk among plastic surgeons (Streu et al. 2013), but not among emergency physicians (Goldberg et al. 2012). Finally, we assessed clinical symptoms with questionnaires and it would be desirable to repeat this assessment with clinical diagnostic interviews.

The present findings have implications for health promotion in ED personnel. Considering the high workload of ED personnel and their growing involvement in hospital admissions, supporting staff who experience flashback memories presents an important public health issue. Burnout and symptoms of psychopathology are associated with suboptimal patient care and absenteeism (Davey et al. 2009; Rosenstein 2013), which present a considerable financial burden as well as causing human suffering. Recent findings that mindfulness and meditation might have a positive impact on physicians’ psychological well-being and capacity for relating to patients (Krasner et al. 2009) raises the possibility that such practices may also be beneficial for ED nurses. Investigating age differences in the success of such programs appear crucial in the light of our findings. In addition, access to regular supervision by trained mental health professionals might help reduce the incidence of intrusive memories by preventing or helping to resolve work conflict and facilitating mental processing of critical incidents, specifically those incidents tied around resuscitation. Such strategies may help prevent the transformation of CIs into “frozen memories” that appear over and over again, in form of intrusive or flashback memories. Future studies should examine to what extent such strategies may help promote the wellness of both younger and older ED personnel who might have had less exposure to the benefits of such interventions during their training. The ultimate aim of such efforts is prevention of flashback memories and increasing the quality of ED healthcare systems.
